# Research into the Bioengineering of a Novel α-Conotoxin from the Milked Venom of *Conus obscurus*

**DOI:** 10.3390/ijms232012096

**Published:** 2022-10-11

**Authors:** Sean Wiere, Christopher Sugai, Michael J. Espiritu, Vincent P. Aurelio, Chloe D. Reyes, Nicole Yuzon, Randy M. Whittal, Jan Tytgat, Steve Peigneur, Jon-Paul Bingham

**Affiliations:** 1 Department of Molecular Biosciences and Bioengineering, College of Tropical Agriculture and Human Resources, University of Hawai’i, Honolulu, HI 96822, USA; 2 School of Pharmacy, Pacific University Oregon, Hillsboro, OR 97123, USA; 3 Department of Chemistry, University of Alberta, Edmonton, AB T6G 2G2, Canada; 4 Toxicology and Pharmacology, University of Leuven (KU Leuven), Campus Gasthuisberg O&N II, 3000 Leuven, Belgium

**Keywords:** α-conotoxin, analog, bioassay, IC_50_, LD_50_, *Conus obscurus*, mass spectrometry, milked venom, post-translational modifications, nAChR

## Abstract

The marine cone snail produces one of the fastest prey strikes in the animal kingdom. It injects highly efficacious venom, often causing prey paralysis and death within seconds. Each snail has hundreds of conotoxins, which serve as a source for discovering and utilizing novel analgesic peptide therapeutics. In this study, we discovered, isolated, and synthesized a novel α3/5-conotoxins derived from the milked venom of *Conus obscurus* (α-conotoxin OI) and identified the presence of α-conotoxin SI-like sequence previously found in the venom of *Conus striatus*. Five synthetic analogs of the native α-conotoxin OI were generated. These analogs incorporated single residue or double residue mutations. Three synthetic post-translational modifications (PTMs) were synthetically incorporated into these analogs: *N*-terminal truncation, proline hydroxylation, and tryptophan bromination. The native α-conotoxin OI demonstrated nanomolar potency in *Poecilia reticulata* and *Homo* *sapiens* muscle-type nicotinic acetylcholine receptor (nAChR) isoforms. Moreover, the synthetic α-[P9K] conotoxin OI displayed enhanced potency in both bioassays, ranging from a 2.85 (LD_50_) to 18.4 (IC_50_) fold increase in comparative bioactivity. The successful incorporation of PTMs, with retention of both potency and nAChR isoform selectivity, ultimately pushes new boundaries of peptide bioengineering and the generation of novel α-conotoxin-like sequences.

## 1. Introduction

*Conus* is the world’s largest genus of marine invertebrates, with ~800 known species inhabiting tropical to subtropical waters across the globe. Cone snails can be classified into three feeding subgroups: piscivores (fish-eating), molluscivores (mollusk-eating), and vermivores (worm-eating). These slow-moving gastropods have evolved over millions of years to develop some of the world’s most neuroactive and toxic peptides, called conotoxins. Delivery of these conotoxins in a prey strike employs a disposable harpoon-like radular tooth that reaches acceleration speeds of up to 400,000 m s^−2^ [1]. The delivered conotoxins can cause paralysis and even death in prey within seconds of injection [2] and have caused up to 36 human deaths [3].

Conotoxins have high isoform selectivity amongst ion channels, receptors, and transporters within the central nervous system [4]. This group of peptides has proven to be valuable as analgesic drug candidates and pharmacological probes, with potential therapeutic implications in pain management, autoimmune disorders, anxiety, depression, and even cancer. Mining the treasure trove of biologically active conotoxins is an attractive venture for research. It is estimated that up to one million theoretical conotoxins exist, with a small but apparent overlap between species [5,6]. Less than 1% of these conotoxins have been sequenced thus far. An even smaller percentage (1–2%) of those currently being sequenced have been characterized pharmacologically [7].

Alpha (α) conotoxins were among the first conotoxins to be discovered and have been extensively studied for their pharmacological properties. α-Conotoxins typically range between 12–20 amino acids (αα) in length and act as competitive antagonists toward the nicotinic acetylcholine receptor (nAChR). These conotoxins can differentiate between several isoforms of neuronal and muscle nAChRs [8]. Within the α-conotoxin superfamily (αA), ~75% possess the cysteine “framework I” of C_a_C_b_-C_c_-C_d_, [7,9] (www.conoserver.org, accessed on 6 September 2022) and display well-defined three-dimensional structures due to the backbone constraints caused by two disulfide [10] bonds. Of these “framework I” peptides, a 3/5 configuration is predominately observed. This configuration contains three αα residues in the first intercysteine loop and five αα in the second. These conotoxins also have an established disulfide connectivity of C_a_-C_c_ and C_b_-C_d_. This connectivity gives rise to 3/5′s characteristic native globular structure.

Post-translational modifications (PTMs), such as disulfide bonding, add a layer of chemical diversity, impacting beyond that of varying traditional residues within the peptide backbone. This increased sequence diversity can affect peptide selectivity, potency, and stability [11]. PTMs are extensively employed within *Conus* as a strategic mechanism to enhance prey immobilization and capture.

**Figure 1:**
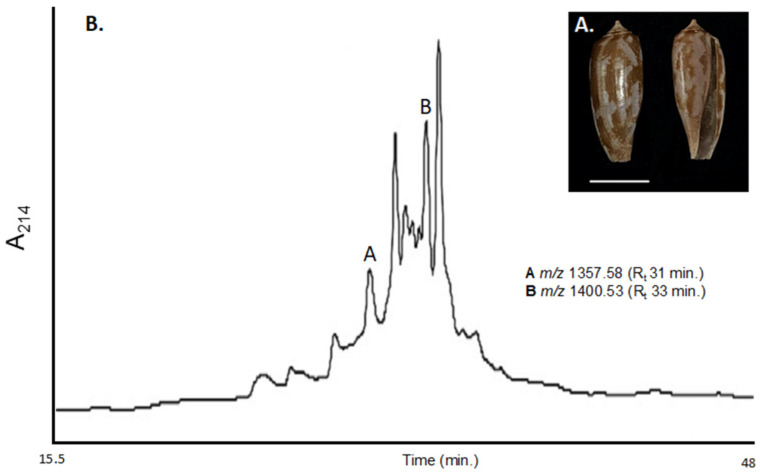
(**A**) *Conus obscurus* shell found off Oahu, Hawai’i, USA. Scale bar 1 cm; (**B**). RP-HPLC/UV profile of the Tris(2-carboxyethyl) phosphine (TCEP) reduced, pooled milked venom from *Conus obscurus* extracted at 214 nm. Peaks corresponding to α-conotoxin SI and α-conotoxin OI are indicated, as well as their observed TCEP reduced *m/z*. TCEP reduction of whole MV increased peak resolution and improved peptide isolation. See Supplementary Figure S1 for total *m/z* observed within the crude TCEP reduced MV.

*Conus obscurus* is a piscivorous cone snail that has not been thoroughly studied, mainly due to the rarity of the snail and low venom yields reflective of the cone snail’s small physical size (Figure 1) [12]. Only ten peptides have been documented thus far from the venom of *C. obscurus*. Of those peptides, half originate from cDNA clones, from which only the primary sequence is derived, requiring further clarification of the presence of PTMs in the mature peptide. Only two conotoxins have been pharmacologically characterized from *C. obscurus* thus far: α-conotoxins OIVA and OIVB. These conotoxins belong to the αA-conotoxin superfamily and demonstrate potency and selectivity towards the fetal neuromuscular nAChR (αβγδ). Interestingly, both show a much lower affinity for the adult neuromuscular nAChR (αβϵδ) and virtually no inhibitory effects on the neuronal nAChRs [13,14].

In this work, we isolated and characterized two αA-conotoxins found in the milked venom (MV) of *Conus obscurus*; one novel (α-conotoxin OI) and one α-conotoxin-like sequence previously documented in another cone snail species (α-conotoxin SI). We also chemically generated several α-conotoxin OI analogs, some of which incorporated novel single and double-point mutations in the form of PTMs. These toxins were then assessed for bioactivity against various neuronal and muscle nAChR isoforms.

## 2. Results

### 2.1. Identification of α-Conotoxins within the Milked Venom of Conus obscurus

Two peaks were isolated via RP-HPLC/UV using a TCEP reduced, pooled MV sample of *C. obscurus*: 1357.58 and 1400.53 Daltons (Da), respectively (Figure 1 and Figure S1). Isolated peptides were sent to the University of Alberta Chemistry Department for sequencing analysis and in-house evaluation (see Supplementary Figures S2 and S3). MALDI TOF analysis confirmed corroborating peaks from both interpreters. Interpreters agreed upon the two *C*-terminally amidated peptides (Table 1).

Sequence 1400.53 Da resembled a novel sequence and was named α-conotoxin OI (α-OI). Sequence 1357.58 Da resembled α-conotoxin SI, previously isolated from *Conus striatus* and described by Zafaralla et al. [15]. Both interpreters agreed that 1357.58 Da was a potential match with α-conotoxin SI, considering the presence of the isobaric ααs Isoleucine and Leucine. Both peptides are considered α3/5-conotoxins with a cysteine framework of X-C_a_-C_b_-3(X)-C_c_-5(X)-C_d_ (X = αα).

### 2.2. Structural Design of the Novel Analogs of α-Conotoxin OI

α-Conotoxins with an intercystiene loop size of 3/5 antagonize the mammalian skeletal muscle-type nAChR. α3/5-conotoxins can also have high specificity for the delta subunit of neuromuscular nAChR (αβδ). From a bioengineering perspective, an examination of each amino acid position in every documented α3/5 sequence provides a better understanding of this paralytic subfamily of conotoxins, specifically the type of amino acids, their relative position(s), their chemical nature/diversity, and their impact on toxicity, dissociation rate, binding affinity, etc.

**Figure 2:**
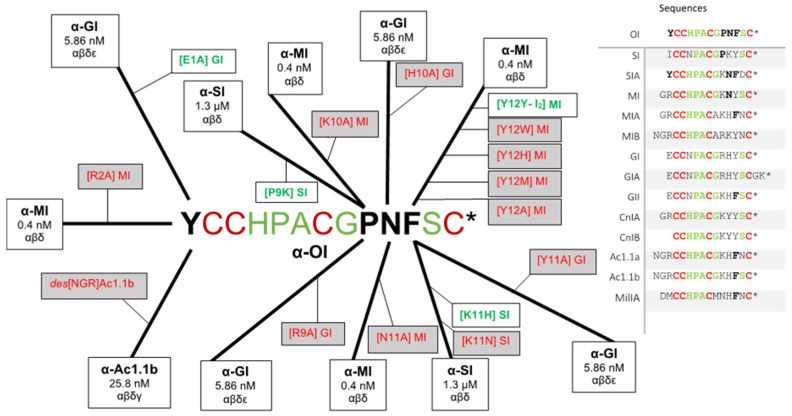
Site-specific mutational map of α3/5′s compared to α-conotoxin OI. Within the α-conotoxin OI peptide sequence, the bold amino acids (αα) represent variable positions, while the green αα are generally conserved (cysteines highlighted in red). The long branch signifies the parent α-conotoxin, while the shorter branch coming off the extended branch is the corresponding synthetic analog. A red analog indicates the mutated conotoxin’s half maximal effective concentration (IC_50_) increased (less toxic), and a green analog indicates the mutated conotoxin’s IC_50_ decreased (more toxic). The number within the α-conotoxin boxes indicates the observed IC_50_ towards the αβδ isoform of the muscle nAChR. Sequences of illustrated α3/5-conotoxins are provided, with regions of conserved αα presented. Asterisk (*) indicates the presence of a *C*-terminal amide.

Amino acid positions 1, 4, and 9–11 have the highest hypervariability in the α3/5-conotoxin sequences. The process of determining *C. obscurus* α-conotoxin analogs is illustrated in Figure 2, which depicts the mind map used to determine the best possible analogs of α-conotoxin OI to increase fish lethality and selective inhibition at mammalian muscle nAChR. To date, the five conotoxins that branch off the parent native α-conotoxin OI are the most bioengineered α3/5-conotoxins. However, numerous other α3/5-conotoxins (25+) were also examined in the determination of α-conotoxin OI analogs.

Positions 9, 10, and 11 in the second disulfide loop are highly variable throughout the α3/5 subfamily and have been shown to be significant in species specificity and the blocking of the muscle-type nAChR [16]. Altogether, four important residue components are relevant to toxicity and affinity in the second disulfide loop: structure, hydrophobicity, polarity, and electrostatic charge. Minor adjustments to these four components may have an imperative influence on conotoxin toxicity/affinity to the mammalian muscle nAChR.

Five analogs of the α-conotoxin OI parent template were developed (Table 2). These analogs contained a single or double-point mutation, incorporating a range of PTMs: proline hydroxylation, *N*-terminal truncation, and tryptophan bromination. Mutations in each analog have a minimum of one *n* loop (second disulfide loop) substitution, some accompanied by an *N*-terminal truncation. Each modification aimed to increase fish lethality and specificity towards the mammalian muscle nAChR.

### 2.3. Synthesis of α-Conotoxin OI and the Five Structural Analogs

**Figure 3:**
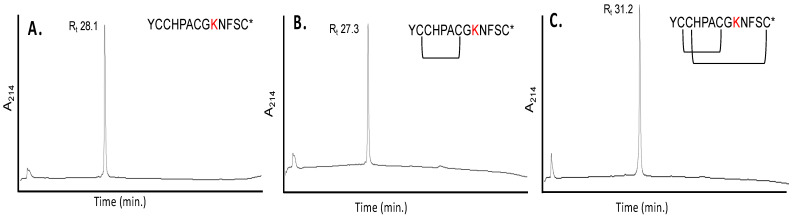
RP-HPLC/UV profiles represent changes in elution time of α-[P9K] conotoxin OI during the sequential two-step oxidation process. (**A**) The synthesized, cleaved, and RP-HPLC/UV purified α-[P9K] conotoxin OI was eluted at 28.1 min. (**B**) Purified α-[P9K] conotoxin OI after first disulfide bond formation using 0.1 M NH_4_HCO_3_, eluting at 27.3 min. (**C**) Purified α-[P9K] conotoxin OI fully oxidized after second disulfide bond formation with iodine, eluting at 31.2 min (see Methods).

α-Conotoxin OI and the five analogs underwent Fmoc-SPPS using a single sequential split-resin approach. The analog α*-des*[Y] [P9O] conotoxin OI was split in half and terminated after the last *N*-terminal cysteine, with the other half completing the addition of the *N*-terminal αα of α-[P9O] conotoxin OI. Each analog underwent two sequential oxidations and was isolated and purified via RP-HPLC once there was an observed shift in retention time. Figure 3 shows an example of α-[P9K] conotoxin OI oxidation and purification with the observed change in retention time upon disulfide bond formation. α*-des*[Y] [P9O] Conotoxin OI yielded low sample yields after iodine oxidation (<40% of the chromatographic UV profile). However, all other analogs and α-conotoxin OI possessed a high yield (>90–95% of chromatographic UV profile).

### 2.4. Fish Bioassay (LD_50_)

α-Conotoxin OI and the five synthetic analogs were administered intramuscularly into *Poecilia reticulata* to determine the median LD_50_. Table 2 illustrates a side-by-side comparison of the different LD_50_ values recorded. The single mutation α-[P9K] conotoxin OI produced the lowest LD_50_ value of 0.95 μg/g (0.67 nMol/g), while α*-des*[Y] [P9O] conotoxin OI had the highest LD_50_ value of 16.25 μg/g (13.00 nMol/g). α-Conotoxin OI and the other three analogs possessed relatively low LD_50_ values, falling from 1.88 to 3.30 μg/g (1.22 to 2.36 nMol/g).

### 2.5. Functional Characterization of Muscle nAChR (IC_50_)

**Figure 4:**
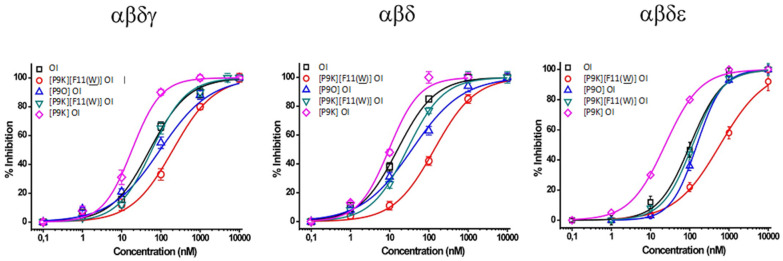
Percent inhibition of *Xenopus laevis* oocytes expressing nAChR isoforms: αβδγ, αβδ, and αβδϵ by α-conotoxin OI and analogs on a nanomolar scale.

α-Conotoxin OI and four analogs were analyzed to determine their half-maximal effective concentration (IC_50_). Due to low oxidative yields, the activity of the analog α*-des*[Y] [P9O] conotoxin OI was not investigated. The following peptides exhibited 100% inhibition (disregarding α-[P9K] [F11(W)] conotoxin OI with αβδϵ) on a nanomolar scale on muscle-type nAChR isoforms: αβδ, αβγ, and αβδϵ (Figure 4). However, none of the following peptides possessed inhibition on a nanomolar scale towards the γ-mammalian muscle (αβγ) nAChR isoform. Moreover, α-conotoxin OI and the four analogs displayed no inhibition to the following human neuronal nAChR isoforms: α3β2, α4β2, α4β4, α7, and α9α10 at >100μM. This trend is common within most α3/5-conotoxins, with the highest specificity towards the αβδ isoform.

According to Table 2, α-[P9K] conotoxin OI exhibited the lowest IC_50_ at 9.6 +/− 1.7 nM towards the αβδ isoform, followed by the native α-conotoxin OI at 16.9 +/− 1.6 nM. However, the other three analogs expressed a higher IC_50_, with the brominated α-conotoxin OI analog resulting in 160.7 +/− 14.7 nM (~9-fold greater than α-conotoxin OI). IC_50_ readings of the following peptides towards the αβγ subunit stoichiometry exhibited concentrations higher than 10 μM. Due to the limited availability of materials, the IC_50_ for the αβγ nAChR isoform was not determined.

The single proline substitution of α-[P9K] conotoxin OI increased the toxicity of the native peptide, as seen in the similar mutation α-[P9K] conotoxin SI via Groebe et al. [17]. However, replacing phenylalanine with tryptophan in α-[P9K] [F11W] conotoxin OI eliminated the effects of the proline substitution. The affinity of these peptides for the αβγδ and αβϵδ nAChR isoforms was also determined. α-Conotoxin OI and its analogs exhibited an overall lower inhibition of the adult nAChR isoform, showing preferred specificity towards the fetal nAChR isoform. The analogs with double point mutations showed an overall decrease in activity against the muscle nAChR isoforms compared to the single mutated analogs. The order of inhibitory response with α-conotoxin OI and its analogs remained the same throughout; αβδ (most inhibited) > αβδγ > αβδϵ > αβγ (least inhibited) isoforms.

## 3. Discussion

### 3.1. α-Conotoxin OI Introduction and Characterization

This paper highlights the discovery of a novel αA-conotoxin in the MV of *Conus obscurus.* An MS-driven approach was used to determine the two sequences within the MV: α-conotoxins SI (like) and the novel α-OI. In the determination of the α-conotoxin SI peptide sequence, the *N*-terminal was inconclusive (I/L) due to isobaric molecular weight. Moreover, the peptide is assumed to contain isoleucine at the *N*-terminus, as found in α-conotoxin SI.

α-Conotoxin OI has two distinct characteristics that set it apart from the other discovered α3/5-conotoxin sequences: An *N*-terminal aromatic αα and Pro-9. Moreover, α-conotoxin SI, initially found in the venom of *C. striatus* in Zafaralla et al. [15], is now “apparently” expressed in *C. obscurus* MV. Determining an evolutionary relationship based upon mature conotoxin sequences is an attractive venture. However, it has not been proven to be an accurate methodology to assess the phylogeny of *Conus*. Further interpretation would be necessary to examine the evolutionary relationship between these two species thoroughly.

α-Conotoxin OI is now the third conotoxin from *C. obscurus* to be functionally characterized. The α-conotoxin OI structure belongs to the α3/5-conotoxin family, showing high specificity towards the muscle nAChR. α3/5-Conotoxins have a narrow sequence spectrum, which is well illustrated. Conclusively, a bioinformatics approach was used to obtain a variety of analogs to increase the potency of α-conotoxin OI towards the muscle type nAChR and fish (see Figure 4 and Table 2) (to be published elsewhere). We have briefly described our approach and logic in Section 3.2 and Section 3.3 (see below). Combined characteristics to determine analogs individualized each αα within homologous α3/5-conotoxins as it relates to toxicity, dissociation rate, and binding affinity to its preferred receptor target. The determination of analogs also integrated three PTMs further to expand the capabilities of the α3/5-conotoxin family using this approach.

α-Conotoxin OI resulted in an LD_50_ of 2.36 nMol/g in *P. reticulata*, causing fish death in all tested concentrations. α-Conotoxin OI also resulted in an IC_50_ of 16.9 +/− 1.6 nM towards the αβδ nAChR isoform, with higher specificity towards the fetal muscle isoforms. Finally, α-conotoxin OI exemplifies the peptide diversity of the many α3/5-conotoxin sequences discovered with a nanomolar affinity towards the muscle-type nAChR.

### 3.2. Structure–Activity Relationship Data and Pharmacological Activity of Single-Point-Mutations within α-Conotoxin OI

Substituting a hydroxylated proline into the α-conotoxin OI sequence (α-[P9O] conotoxin OI) was the first PTM introduced into analog development. The inclusion of this prevalent PTM was due to certain α4/7 (α-conotoxins EI, α-conotoxin SrIA, and α-conotoxin SrIB) and α4/4 (α-conotoxin EIIA, α-conotoxin EIIB, and α-conotoxin PIB) conotoxins containing hydroxyproline with muscle nAChR affinity, as well as hydroxyproline discovery in α3/5 [Hyp-4] CnID and [Hyp-7] CnIK conotoxins via MS/MS analysis (Table 3A) [18]. The α4/7 and α4/4-conotoxins generally have a high affinity for the neuronal nAChR. However, most α4/7- and α4/4-conotoxins that contain hydroxyproline have muscle-type nAChR affinity. Although conotoxins α4/7 SrIA/B both contain tyrosine in the second disulfide loop and α-conotoxin EI contains conserved histidine and proline in the first disulfide loop. Conotoxins α4/4, α-EIIA, α-EIIB, and α-PIB contain the conserved H/NPA in the first disulfide loop. These characteristics (underlined in Table 3A) are homologous to α3/5-conotoxins and may contribute to muscle nAChR specificity rather than the presence of hydroxyproline [19,20]. Overall, hydroxyproline has been observed in α-conotoxins with muscle nAChR affinity. Whether this has biological relevance or function remains to be established.

The addition of the hydroxyl group in α-[P9O] conotoxin OI retained lethality in fish and increased the IC_50_ ~2-fold towards the αβδ and αβδγ isoforms when compared to the native α-conotoxin OI. This modification thus provides an interesting molecular determinant for potential target differentiation and selectively. A common characteristic of α4/7 and α4/4 shows the presence of hydroxyproline in the *N*-terminal or first disulfide loop [as seen in Table 3A]. However, the PTM in α-[P9O] conotoxin OI is presented in the second disulfide loop. Considering the native α3/5-conotoxins, α-[Hyp-4] CnID and α-[Hyp-7] CnIK with hydroxyproline found in the first disulfide loop, substitution with the proline found in the first disulfide loop of α-conotoxin OI should be examined.

The second PTM introduced, *N*-terminal truncation, was observed in α*-des*[Y] [P9O] conotoxin OI. Truncated peptides present a more drug-like appearance with an economic advantage in producing novel therapeutics [29]. Although, there have been little to no cases of decreased IC_50_ from a synthetic deletion of the entire *N*-terminal in α3/5-conotoxins. Previous research has been unsuccessful in determining the purpose of *N*-terminal deletion in native peptides. Despite this, there are multiple examples of α3/5 *N*-terminal deletion found in nature; α-conotoxin MIC (*Conus magus*) [30], α-des-Ile-conotoxin SI (*C. striatus*) [15], conotoxins α-CnIB/C/D/E/G (*Conus consors*) [18].

After both oxidations, the analog α-*des*[Y] [P9O] conotoxin OI produced a low yield, limiting the analysis to only the fish bioassay. The overall LD_50_ of the truncated analog was 13.00 nMol/g; other analogs plus α-conotoxin OI fell in the range of 0.67 to 2.36 nMol/g.

α-[P9K] conotoxin OI was introduced because of the positive effects of the lysine in this position as shown in past research. Groebe et al. [17] changed Pro-9 to Lys-9 in α-conotoxin SI, producing an 870-fold increase in affinity for the αβδ isoform in mouse cell (BC3H-1) receptors and a 190-fold increase in affinity for the αβγ isoform of *Torpedo californica* receptors. Groebe et al., suggested an imbalance of charge contributed to lowered toxicity. When the proline is replaced with a lysine, this will cause a positive *N*/*C*-terminal, relating the peptide to the other highly toxic α3/5 conotoxins [17]. However, Jacobsen et al. [28] disagree with Groebe et al., stating the proline in α-conotoxin SI displaces the tyrosine in the second disulfide loop out of its optimal position. However, this proline is seen to be passed down throughout the *Conus* genus and ultimately increases the peptide diversity of α3/5-conotoxins.

α-[P9K] conotoxin OI was the only analog that overall decreased the IC_50_ of *H. sapiens* muscle nAChR; ~2-fold in αβδ, ~3-fold in αβδγ and ~4-fold in αβδϵ isoforms. α-[P9K] conotoxin OI also had the lowest LD_50_ characterization at 0.67 nMol/g. This is the second example of proline to lysine substitution increasing the toxicity of the α3/5, as seen in α-[P9K] conotoxin SI. Ultimately, α-[P9K] conotoxin OI favors the suggestion of the negative effect proline may have in this position on the mammalian/fish muscle-type nAChRs.

### 3.3. SAR Data and Pharmacological Activity of Double-Mutated Analogs of α-Conotoxin OI

α-[P9K] [F11W] Conotoxin OI retains the lysine in position 9 and substitutes the aromatic αα phenylalanine for tryptophan. Due to past research, the tryptophan substitution was presumed to have little to no effect on the overall IC_50_ for the mammalian muscle nAChR or fish lethality. The only synthetic tryptophan substitution in α3/5 conotoxins was completed via Jacobsen et al. [28]. The substitution α-[Y12W] conotoxin MI maintained high affinity towards the αβδ mouse muscle nAChR isoform, only increasing the IC_50_ from 0.40 +/− 0.17 nM to 1.5 +/− 0.2 nM [28]. However, tryptophan has not been found in position 11 in native α3/5-conotoxins.

α-[P9K] [F11W] conotoxin OI IC_50_ results proved a level of contrast, as changing an aromatic αα within this position significantly increased the *H. sapiens* muscle nAChR. The IC_50_ increased ~3-fold towards αβδ/αβδγ and ~5-fold towards αβδϵ isoforms when compared to α-[P9K] conotoxin OI. In other words, the phenylalanine to tryptophan substitution eliminated the positive effects of the lysine in position 9 towards the *H. sapiens* muscle nAChR. α-[P9K] [F11W] Conotoxin OI resulted in having a higher IC_50_ than the native α-conotoxin OI peptide in the αβδ, αβδγ and αβδϵ isoforms but did have increased toxicity in fish.

As illustrated in Table 3B, α-[Y12W] conotoxin MI had a 1.1 nM (~4-fold) IC_50_ increase to α-conotoxin MI and was considered overall to have no significant change in potency [28]. Since α-[P9K] conotoxin OI had a higher relative concentration, the IC_50_ comparison of α-[P9K] conotoxin OI and α-[P9K] [F11W] conotoxin OI is comparatively significant at an 18.5 nM increase, even if we had a lower fold increase. Past research indicates that the αα position 11’s aromaticity, hydrophobicity, and bulkiness anchor the peptide into the hydrophobic pocket in the muscle nAChR. Tryptophan is the largest αα, containing a bulky bicyclic aromatic indole ring composed of one benzene and pyrrole group, which accounts for its high hydrophobicity. Although tyrosine and phenylalanine only have one benzene ring, tryptophan’s pyrrole group may disrupt peptide/receptor binding, thus affecting the IC_50_ of α-[P9K] [F11W] conotoxin OI towards muscle nAChR. Our research highlights position 11′s geometric structural importance in retaining mammalian muscle nAChR affinity, compounding the apparent native absence of tryptophan in this position.

The last PTM tested was the bromination of tryptophan, represented in the double mutated analog α-[P9K] [F11(W)] conotoxin OI. L-6-bromotryptophan is found natively throughout *Conus* venom, and the overall purpose of this PTM in nature is poorly understood [11]. Our choice of L-5-bromotryptophan was two-fold: (i) to extend the PTM variability in conotoxin bioengineering and (ii) to examine its impact on biological activities. This initial examination aligns with an assessment of other PTM isomers, as seen with *cis-* and *trans*-hydroxyproline [31].

The rationale for bromotrytophan introduction into α-conotoxin OI was, in part, due to the increased toxicity recorded by the addition of monoiodination and diiodination of tyrosine in α-conotoxin MI by Luo et al. [32] and Jacobsen et al. [28]. Luo et al. suggested the addition of the single iodine to tyrosine in position 12 may strengthen hydrophobic interactions with key residues in the αβδ isoform. Jacobsen et al. suggested that by adding diiodine to tyrosine in position 12, the increase in toxicity showed a relatively nonspecific hydrophobic interaction could be involved between the conotoxin and δ receptor interface. Due to the high favorability of a bulky, hydrophobic αα in this position, the addition of L-5-bromotryptophan in α-conotoxin OI in position 11 allowed us to examine any effects of halogenation and non-native αα integration, as well as the PTM bromination of tryptophan, respectively.

α-[P9K] [F11(W)] conotoxin OI had the highest IC_50_ (overall nanomolar affinity still observed) out of all the peptides tested towards αβδϵ and αβδγ *H. sapiens* muscle nAChR isoforms and the synthetic construct αβδ. This result is most likely due to the adverse effects of tryptophan in this position (as shown in α-[P9K] [F11W] conotoxin OI) and reiterates the importance of αα geometry in position 11 for peptide/receptor affinity towards the mammalian muscle nAChR. However, α-[P9K] [F11(W)] conotoxin OI had the second-lowest LD_50_ reading at 1.22 nMol/g in the fish bioassay. The PTM bromination of tryptophan may have evolved into *Conus* venom based on its high potency for marine organisms rather than the mammalian muscle nAChR. There is no shortage of bromine in marine environments as it is a common element in seawater. Brominated compounds have been frequently seen in *Conus* and in sessile marine organisms that use brominated compounds as metabolites for predator defense [33]. Further research and interpretation would be needed to confirm this hypothesis.

## 4. Experimental Procedures

### 4.1. Venom Extraction and Analysis

*C. obscurus* was housed in a modified Aquatic Habitat^TM^ (Apopka, FL, USA) system developed by Bergeron et al. [34] and milked for its venom [12,35]. Pooled MV was desalted using previously established methods (250 total, typically ~1–2 µL/milking) via preparative reversed-phase high-performance liquid chromatography (RP-HPLC) (Vydac, Hesperia, CA, USA; C_18_, 10 μm, 300 Å, 22 × 250 mm) with a 5 mL/min isocratic flow of 0.1% *v/v* TFA. (aq.) (Solvent A) followed by 90/10% *v/v* MeCN/0.08% *v/v* TFA (aq.) (Solvent B). Elution was monitored at 214 nm, collected, freeze-dried, and then reconstituted in Solvent A for storage at −20 °C.

### 4.2. Chromatographic Separation and Analysis

RP-HPLC/UV: Native and synthetic conotoxins were individually separated as follows: (i) Capillary Scale (Phenomenex, Torrance, CA, USA; C_18_, 5 μm, 300 Å, 1.0 × 250 mm, flow 100 μL/min)—used for comparative RP-HPLC/UV profiling, for peptide purity quality control, to quantify peptides, and to perform peptide co-elution experiments. (ii) Analytical Scale (Vydac, Hesperia, CA, USA; C_18_, 5 μm, 300 Å, 4.6 × 250 mm, flow 1 mL/min)—used to isolate and purify native peptides for MS analysis. (iii) Preparative Scale (Vydac, Hesperia, CA, USA; C_18_, 10 μm, 300 Å, 22 × 250 mm, flow 5 mL/min)—used for the preparative separation of crude synthetic peptides for co-elution experiments, structure determination, and pharmacological assays. Systems (i) and (ii) used a Waters 2695 Alliance RP-HPLC System interfaced with a 996 Waters PDA UV Detector (Waters Corporation, Milford, MA, USA) for automated sample analysis and detection. Data were acquired and analyzed using Waters Millennium^32^ (v3.2) software. Samples were eluted using a linear 1%/min gradient of organic Solvent B against aqueous Solvent A for 65 min, excluding a terminating high organic wash (80% Solvent B for 10 min), and a pre-equilibration step (5% Solvent B) for 5 min prior to sample injection. The eluent was monitored from 200–300 nm and extracted at 214 nm. The preparative RP-HPLC/UV system (iii) used a 625 Waters HPLC pump and controller interfaced with a 996 Waters PDA Detector (Waters Corporation, Milford, MA, USA). Both solvent gradient and data acquisition were controlled using the Waters Millennium^32^ (v3.2) software. Filtered (Nylon 0.22 μm) synthetic peptides and crude venom peptide extracts were manually loaded and eluted from the preparative scale column using the same 1% gradient at 5 mL/min and monitored at 214 and 280 nm. Fractions were collected manually and stored at −20 °C or freeze-dried until required.

### 4.3. Mass Spectrometry (MS) Techniques and Peptide Sequencing

A pooled and desalted MV sample was subjected to electrospray ionization mass spectrometry (ESI-MS) to determine the presence of known and unknown molecular masses. The Conoserver (www.conoserver.org) database was consulted for known *C. obscurus* peptides and their parent masses. From these results, the RP-HPLC/UV peaks correlating to α-conotoxins SI and OI were chosen for purification in both native and tris(2-carboxyethyl) phosphine (TCEP) reduced states and were analyzed by matrix-assisted laser desorption/ionization time-of-flight (MALDI-TOF; Bruker Daltonics Inc., Fremont, CA, USA) by multiple interpreters.

A MaXis Impact Q-TOF mass spectrometer interfaced with a Michrom Advance Nano LC (Bruker Daltonics Inc., Fremont, CA, USA) was later used to collect additional LC-MS/MS data on the pooled MV. In addition, ~5% of the isolated native peptide samples underwent ESI-MS analysis through direct injection into an AB/MDS Sciex API 3000 triple quadrupole mass spectrometer (Sciex, Toronto, ON, Canada). In addition, the desalted TCEP reduced peptides (200 mM TCEP, 200 mM NH_4_OAc, pH 4.5, 60 °C for 10 min) were analyzed on a Bruker nanoLC-AmaZon speed electron transfer dissociation (ETD) ion trap MS system (Bruker Daltonics Inc., Fremont, CA, USA) and separated via C_18_ analytical column. The resulting sequence fragmentation data were acquired in ETD and collision-induced dissociation (CID) mode.

A Bruker Ultraflex III MALDI-TOF instrument (Bruker Daltonics Inc., Fremont, CA, USA) was used with Compass 1.2 SR1 software to collect additional sequence data on purified TCEP-reduced native peptides. In addition, as an independent confirmatory measure, MALDI-TOF and FlexAnalysis v3.0 (Bruker Daltonics Inc., Fremont, CA, USA) were used to reconfirm peptide sequences.

### 4.4. Peptide Synthesis

Confirmation of the α-conotoxin OI sequence allowed for the construction of the native-like peptide and five analogs via 9-Fluorenylmethoxycarbonyl solid-phase peptide synthesis (Fmoc-SPPS). Synthesis of contrived linear peptides accompanied by standard αα side-chain protection, with all cysteine residues being trityl-protected, was undertaken. Fmoc αα (4-fold excess; 2 mM) were assembled on a CLEAR-Amide^TM^ resin (0.5 mM; Vivitide, Louisville, KY, USA) at 0.47 meq/g. Coupling times were 15 min for each cycle, except for Fmoc-5-Bromo-L-tryptophan, which was coupled with 2.3 mM for 60 min.

The general synthesis protocol was adapted by Kapono et al. [30]. In brief, CLEAR-Amide^TM^ resin (Vivitide, Louisville, KY, USA) was swelled and shaken in 4 mL of dimethylformamide (DMF) overnight. The resin was washed with DMF (3× 20 mL), deprotected with 50% (*v*/*v*) Methyl-piperidine in DMF (2× 5 mL), and rewashed with DMF (3× 20 mL). Fmoc αα (4-fold molar excess) were activated in-situ via dissolving in HBTU/DMF (4 mL) and activating with 347 µL (2 mM) *N,N*-Diisopropylethylamine (DIEA). The activated αα was then coupled to the resin for 15 min. After coupling, a ninhydrin test was performed [36], using a NanoDrop^TM^ ND-1000 UV spectrophotometer (Thermo Fisher. Waltham, MA, USA) to determine the percent coupling of the αα to the resin. Once the percent coupling was ≥99.5%, the peptidyl-resin was washed thoroughly with DMF and *N-*terminally deprotected. As stated above, the in-situ activation of the next αα in the peptide sequence was then undertaken and resin-coupled. Once the complete peptide sequence was constructed from the *C-*terminus to the *N*-terminus, the *N*-terminus was finally deprotected and washed with DMF (3× 20 mL), then with dichloromethane (DCM) (5× 20 mL), followed by drying under N_2_.

The resulting peptidyl-resin was cleaved using Reagent K (82.5% *v*/*v* TFA, 5% *v*/*v* phenol, 5% *v*/*v* deionized (DI) water, 5% *v*/*v* thioanisole, 2.5% *v*/*v* 1,2-ethanedithiol) in a ratio of 30:1 mL/g peptidyl-resin for 2 h. The resin was filtered, with the resulting dissolved peptide precipitated by adding liquid N_2_ chilled tert-butyl methyl ether (10–20 mL). The peptide precipitate was pelleted by centrifugation (3000× *g*, 4 °C, 10 min) and washed with chilled tert-butyl methyl ether. The ether wash and pelleting process were repeated twice. The resulting peptide pellet was suspended in 25% *v*/*v* acetic acid and freeze-dried. The peptide was then directly subjected to oxidation.

### 4.5. Peptide Oxidation

Oxidation differs between α-conotoxin OI and the five analogs. α-Conotoxin OI contained 4 S-trityl protected cysteines (C_a_, C_b_, C_c_ and C_d_), enabling random disulfide folding once cleaved. α-Conotoxin OI analogs had 2 S-trityl (C_a_–C_c_), and two acetamidomethyl (Acm) (C_b_–C_d_) protected cysteines to obtain the globular isomer structure found natively in α-OI (C_a_–C_c_ and C_b_–C_d_).

α-Conotoxin OI was oxidized first to confirm the preferred isomer structure. Most α3/5-conotoxins take on the globular isomer structure with consistency in RP-HPLC profile representation—a prominent globular peak with minor tailing isomers (not shown).

α-Conotoxin ObI was placed under nine different oxidation conditions with various components, pH, temperature, and durations (see Supplementary Table S1). The condition with the highest yield (>90–95% relative UV peak height) of the preferred globular isomer was analyzed through RP-HPLC analysis. The highest yield for the globular α-conotoxin OI was achieved using 0.1 M ammonium bicarbonate, pH 8, 25 °C, for 5 days at a 1:10 ratio (*w*/*v*). Upon completion, materials were acidified dropwise with 100% acetic acid until a pH of 4 was reached, then directly desalted via semi-preparative RP-HPLC/UV and freeze-dried. Finally, the material was purified via analytical RP-HPLC/UV, and ESI-MS confirmed the monoisotopic target mass (MH^+^).

Five analogs of α-conotoxin OI underwent selective disulfide folding. The first disulfide oxidation using the above conditions formed the first disulfide bond (C_a_–C_c_). The partially oxidized material underwent spontaneous deprotection (Acm removal) and oxidation, forming the 2nd disulfide bond (C_b_–C_d_) using iodine. Here, iodine crystals (21 mg) were dissolved in 840 µL of 100% acetic acid; 600 µL of this solution was added to 1–5 mg of peptide, dissolved in 2 mL of 50% *v/v* acetic acid. The peptide–iodine solution was vortexed for 5 min and quenched with the dropwise addition (4 µL) of 1 M sodium thiosulfate (Na_2_S_2_O_3_) until the solution was colorless. The resulting mixture was quenched by adding 5–7 mL of Solvent A. The fully oxidized peptide was purified and desalted, as above. ESI-MS was used to confirm the MH^+^ before and after each oxidation step.

### 4.6. Amino Acid Analysis

Once oxidized, RP-HPLC/UV purified and freeze-dried, a sample of each peptide was sent to the UC Davis Molecular Structure Facility to undergo αα analysis. Peptides were quenched 2× 200 µL formic acid/MeCN and transferred (entire sample) to liquid phase hydrolysis (200 µL 6N HCl/1% phenol at 110 °C for 24 h). Once hydrolyzed, the sample was dissolved in NorLeu dilution buffer and vortexed/spun down. A total of 50 µL of the sample was loaded into an L-8800 Hitachi αα analyzer (Hitachi, Tokyo, Japan). Ion-exchange chromatography was used to separate αα, followed by a “post-column” ninhydrin reaction detection system to quantify individual ααs. Peptides were presented in [nMol/µL].

### 4.7. Fish Bioassay (LD_50_)

α-Conotoxin OI and its analogs were injected into fish (*Poecilia reticulata*) in triplicate to determine their median lethal doses (LD_50_). Peptide doses (D) ranged from 20, 10, 5, 2.5, and 1 nMol/g of fish (0.11–0.20 g) in 1× phosphate-buffered saline (PBS). Via a Hamilton microliter syringe, 5 µL injections were administered intramuscularly into the fish. Methods were adapted from Meier et al. [37] to reduce experimental animal numbers.

Survival times (T) were recorded in seconds from injection to observed death. D (μg/g) was plotted versus D/T [(μg/g)/seconds], with the resulting linear regression (y = *a*x + *b*) used to calculate the LD_50_, which is equal to the y-intercept (b) [37].

### 4.8. Functional Characterization (IC_50_)

#### 4.8.1. Expression of Voltage-Gated Ion Channels in *Xenopus laevis* Oocytes

Stoichiometry of subunit constructs for oocytes was (i) human isoform αβδϵ and the synthetic constructs αβδ, αβγ, and (ii) human neuronal nAChR isoforms: α3β2, α4β2, α4β4, α7, and α9α10, in *Xenopus* oocytes, the linearized plasmids were transcribed using the T7 or SP6 mMESSAGE-mMACHINE transcription kit (Ambion^®^, Carlsbad, CA, USA). Previously described methods for harvesting stage V–VI oocytes from anesthetized female *Xenopus laevis* frogs [38] were used. Oocytes were injected with 50 nL of cRNA at a concentration of 1 ng/nL using a micro-injector (Drummond Scientific^®^, Broomall, PA, USA). The oocytes were incubated in a solution containing (in mM): NaCl, 96; KCl, 2; CaCl_2_, 1.8; MgCl_2_, 2; and HEPES 5 (pH 7.4), supplemented with 50 mg/L gentamicin sulfate.

#### 4.8.2. Electrophysiological Recordings

Two-electrode voltage-clamp recordings were performed at room temperature (18–22 °C) while using a Geneclamp 500 amplifier (Molecular Devices^®^, Downingtown, PA, USA) controlled by a pClamp data acquisition system (Axon Instruments^®^, Union City, CA, USA). Whole-cell currents from oocytes were recorded 1–4 days after injection. The bath solution composition was (in mM): NaCl, 96; KCl, 2; CaCl_2_, 1.8; MgCl^2^, 2; and HEPES 5 (pH 7.4). Voltage and current electrodes were filled with 3 M KCl. The resistances of both electrodes were kept between 0.7 and 1.5 MΩ. During recordings, the oocytes were voltage-clamped at a holding potential of −70 mV and continuously superfused with ND96 buffer via gravity-fed tubes at 0.1–0.2 mL/min with a 5 min incubation time for the bath-applied peptides. Acetylcholine (ACh) was applied via gravity-fed tubes until peak current amplitude was obtained (1–3 s), with 1–2 min washout periods between applications. The nAChRs were gated by a variable time-duration pulse of ACh (200 µM for α1β1γδ, α4β2, α4β4, α1β1δε; 100 µM for α7; and 500 µM for α9α10) for the different nAChR subtypes at 2 mL/min. Data were sampled at 500 Hz and filtered at 200 Hz. Peak current amplitude was measured before and after the peptide incubation.

To assess the concentration–response relationships, data points were fitted to the Hill equation: y = 100/[1 + (IC_50_/[toxin])h], where y is the amplitude of the toxin-induced effect, IC_50_ is the toxin concentration at half-maximal efficacy, [toxin] is the toxin concentration, and h is the Hill coefficient. A comparison of two sample means was made using a paired Student’s *t*-test (*p* < 0.05). The data is presented as the mean ± standard error (SEM) of at least six independent experiments (*n* ≥ 6). Data was tested for normality using a D’Agostino–Pearson omnibus test, with variance tested using the Bonferroni or Dunn’s test. Data following a Gaussian distribution were analyzed for significance using a one-way variance analysis (ANOVA). Non-parametric data were analyzed for significance using the Kruskal–Wallis test. Differences were considered significant if the probability that their difference stemmed from chance was <5% (*p* < 0.05). Data were analyzed using pClamp Clampfit 10.0 (Molecular Devices^®^, Downingtown, PA, USA) and Origin 7.5 software (Originlab^®^, Northampton, MA, USA).

## 5. Conclusions

Our research investigated the native sequence discovery of *Conus obscurus* α-conotoxins via MS analysis and the pharmacological characterization of these peptides. After determining the sequence of the peptides, we used a bioinformatics approach to develop five analogs based upon the parent α-conotoxin OI sequence successfully. These peptides cause fish lethality at nanomolar dosages. Four peptide analogs also retained nanomolar affinity towards the *H. sapiens* muscle-type nAChR. We have also expanded the diversity within the α3/5 cysteine framework by incorporating PTMs into these conotoxins. This work expands upon the α-conotoxin’s ability to achieve prey immobilization and furthers the drive for peptide bioengineering in pharmaceutical research and development.

## Figures and Tables

**Table 1:** Peptide sequence comparison of analogs and native α-conotoxins discovered within the milked venom of Conus obscurus. (A) αα sequences were generated and confirmed via MALDI-TOF sequencing from the pooled TCEP reduced MV of C. obscurus. Molecular masses are calculated in reduced form. α-SI N-terminal contains the isobaric mass corresponding to either isoleucine or leucine. (B) Peptide analogs derived from α-OI. Molecular masses are calculated in the oxidized form (disulfide bonds present). The αα highlighted in red represent single and double mutations introduced into α-OI.

	α-Conotoxin	Sequence	Molecular Mass (Da)
(A)	SI	(I/L)**CC**NPA**C**GPKYSC*	1357.6 (linear)
	OI	Y**CC**HPA**C**GPNFSC*	1400.5 (linear)
(B)	[P9O] OI	Y**CC**HPA**C**GONFSC*	1412.5
	*des*[Y] [P9O] OI	**CC**---**C**-O---C*	1249.4
	[P9K] OI	-**CC**---**C**-K---C*	1427.5
	[P9K] [F11W] OI	-**CC**---**C**-K-W-C*	1466.6
	[P9K] [F11(W)] OI	-**CC**---**C**-K-(W)-C*	1545.5

(W), 5-Bromotryptophan; *, *C*-terminal amidation; *des*[Y], *N*-terminal truncation; O, 4-*trans*-hydroxyproline.

**Table 2:** Pharmacological Activity of α-conotoxin OI and analogs.

α-Conotoxin		Pharmacological Activity (IC_50_)			
	LD_50_ (Fish) (nMol/g)	αβδ (nM)	αβγ (μM)	αβγδ (nM)	αβϵδ (nM)
OI	2.4	16.9 ± 1.6	>10	52.1 ± 6.6	102.8 ± 12.5
[P9O] OI	2.0	37.1 ± 4.9	>10	95.3 ± 5.7	152.1 ± 2.1
*des*[Y] [P9O] OI	13.0	ND	ND	ND	ND
[P9K] OI	0.7	9.6 ± 1.7	>10	18.4 ± 1.8	23.6 ± 1.2
[P9K] [F11W] OI	1.5	28.1 ± 2.9	>10	59.1 ± 5.7	118.6 ± 7.7
[P9K] [F11(W)] OI	1.2	160.7 ± 14.7	>10	202.5 ± 29.1	591.8 ± 50.5

(W), 5-Bromotryptophan; *des*[Y], *N-*terminal truncation; ND, Not Determined; O, 4-*trans*-hydroxyproline.

**Table 3:** Hydroxyproline and tryptophan PTMs and their substitution in α-conotoxins that have muscle nAChR affinity. (A) Comparison between α-[P9O] OI and α3/5, α4/4, and α4/7 sequences with muscle nAChR specificity that contain hydroxyproline. The cysteines are in bold, hydroxyprolines are highlighted in red, and homologous αα in relation to α3/5 conotoxins are underlined. (B) Comparison of pharmacological activity of tryptophan substitution in α-MI and α-OI. The cysteines are in bold, and the position of mutation is highlighted.

	α-Conotoxin	Loop Size	Sequence	Target	PA (nM)	Organism	Ref.
(A)	[P9O] OI	**3/5**	Y**CC**HPA**C**GONFSC*	αβδ	37.1 ± 4.9	*Homo sapiens*	This work
	[Hyp-4] CnID	**3/5**	**CC**HOA**C**GKHFNC*	ND	ND	ND	[18]
	[Hyp-7] CnIK	**3/5**	NGR**CC**HOA**C**GKYYSC*	ND	ND	ND	[18]
	EIIA	**4/4**	ZTOG**CC**WNPA**C**VKNRC*	αβγδ	0.46 ± 0.15	*Torpedo marmorata*	[21]
	EIIB	**4/4**	ZTOG**CC**WHPA**C**GKNRC*	αβγδ	2.2 ± 0.7	*Torpedo marmorata*	[22]
	PIB	**4/4**	ZSOG**CC**WNPA**C**VKNRC*	αβϵδ	36	*Mus musculus*	[23]
	EI	**4/7**	RDO**CC**YHPT**C**NMSNPQIC*	αβϵδ	65.9 ± 15.7	*Mus musculus*	[24]
	[O3A] EI	**4/7**	RDA**CC**YHPT**C**NMSNPQIC*	αβϵδ	104 ± 28	*Mus musculus*	[24]
	SrIA	**4/7**	RT**CC**SROT**C**RMγYPγL**C**G*	αβγδ	ND	*Homo sapiens*	[23]
	SrIB	**4/7**	RT**CC**SROT**C**RMEYPγL**C**G*	αβγδ	ND	*Homo sapiens*	[23]
	[γ15E] SrIB	**4/7**	RT**CC**SROT**C**RMEYPEL**C**G*	αβγδ	1.8 ± 1.9	*Homo sapiens*	[23]
	Vc1A	**4/7**	G**CC**SDOR**C**NYDHPγIC*	αβγδ	NA	*Rattus norvegicus*	[25]
	[P9O] Vc1.1	**4/7**	G**CC**SDOR**C**NYDHPEIC*	αβγδ	NA	*Rattus norvegicus*	[25]
	GID	**4/7**	IRDγ**CC**SNPA**C**RVNNOHVC*	αβγδ	NA	*Rattus norvegicus*	[26]
	ArIA	**4/7**	IRDE**CC**SNPA**C**RVNNOHV**C**RRR*	αβγδ	NA	*Rattus norvegicus*	[27]
(B)	[P9K] OI	**3/5**	Y**CC**HPA**C**GKNFSC*	αβδ	9.6 ± 1.7	*Homo sapiens*	This work
	[P9K] [F11W] OI	**3/5**	Y**CC**HPA**C**GKNWSC*	αβδ	28.1 ± 2.9	*Homo sapiens*	This work
	MI	**3/5**	GR**CC**HPA**C**GKNYSC*	αβδ	0.40 ± 0.17	*Mus musculus*	[28]
	[Y12W] MI	**3/5**	GR**CC**HPA**C**GKNWSC*	αβδ	1.5 ± 0.2	*Mus musculus*	[28]

*, *C*-terminal amidation; O, 4-*trans*-hydroxyproline; Z, pyroglutamic acid; γ, γ-carboxyglutamate; NA, not active; ND, not determined; PA, pharmacological activity.

## Data Availability

Not applicable.

## Supplementary Materials

The supporting information can be downloaded at: https://www.mdpi.com/article/10.3390/ijms232012096/s1.

## Abbreviations

αα, amino acid; ACh, acetylcholine; Acm, acetamidomethyl; ANOVA, analysis of variance; BC3H-1, mouse cell; CID, collision induced dissociation; D, dosage; Da, Dalton; DCM, dichloromethane; DI, deionized; DIEA, *N,N*-Diisopropylethylamine; DMF, dimethylformamide; IC_50_, half maximal effective concentration; ESI-MS, electrospray ionization mass spectrometry; ETD, electron transfer dissociation; Fmoc-SPPS, 9-Fluorenylmethoxycarbonyl solid phase peptide synthesis; IC_50_, half maximal inhibitory concentration; LC, liquid chromatography; LD_50_, median lethal dose; MALDI-TOF, matrix assisted laser desorption/ionization time of flight; MeCN, acetonitrile; MH^+^, monoisotopic mass; MS, mass spectrometry; MV, milked venom; Na_2_S_2_O_3_, sodium thiosulfate; nAChR, nicotinic acetylcholine channel receptor; NH_4_HCO_3_, ammonium bicarbonate; PBS, phosphate-buffered saline; PDA, photodiode array; PTM, post-translational modification; RP-HPLC, reverse phase high performance liquid chromatography; R_t_, retention time; SEM, mean ± standard error; T, survival times TCEP, tris(2-carboxyethyl) phosphine; TFA, trifluoroacetic acid; UV, ultraviolet; αA, α-conotoxin superfamily; αβγ, gamma neuromuscular nAChR; αβγδ, fetal neuromuscular nAChR; αβδ, delta neuromuscular nAChR; αβϵδ, adult neuromuscular nAChR.
