# Supplementary material for: Research into the Bioengineering of a Novel α-Conotoxin from the Milked Venom of Conus obscurus
Source: Int J Mol Sci. 2022 Oct 11;23(20):12096. doi: 10.3390/ijms232012096 (PMC9602734; doi:10.3390/ijms232012096)

## Slide 1
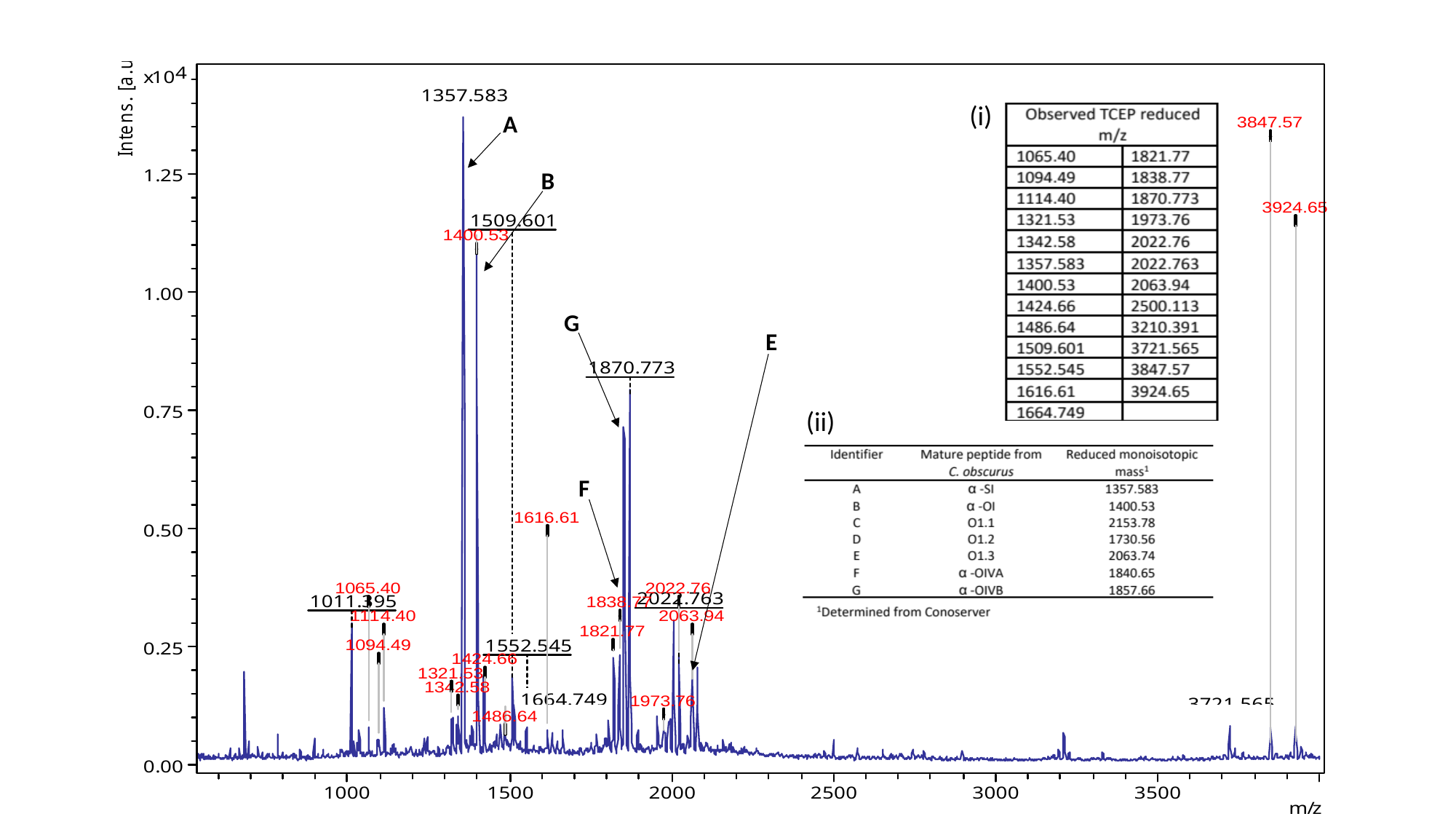

(i)
A
B
G
E
(ii)
F

## Slide 2
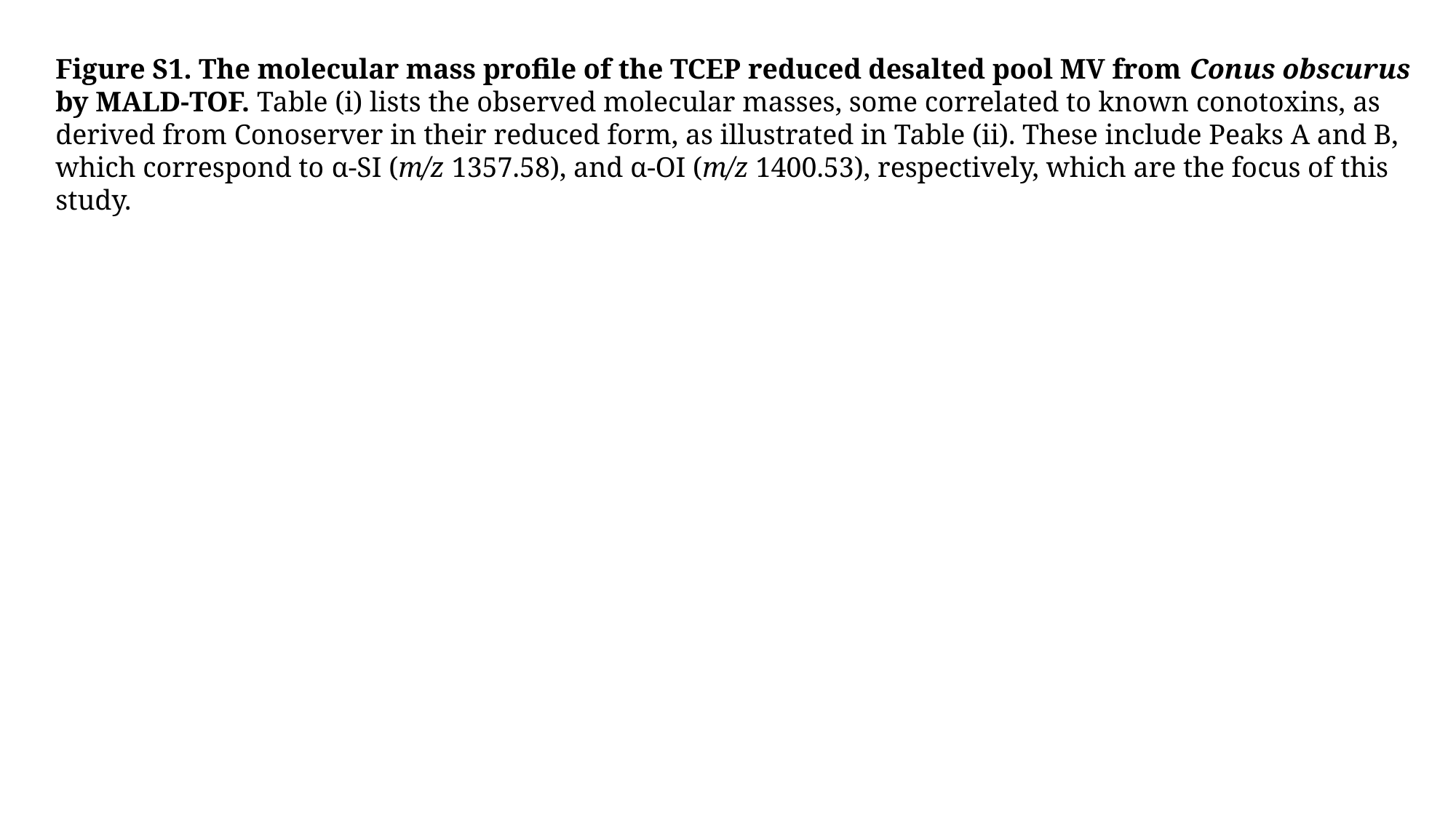

Figure S1. The molecular mass profile of the TCEP reduced desalted pool MV from Conus obscurus by MALD-TOF. Table (i) lists the observed molecular masses, some correlated to known conotoxins, as derived from Conoserver in their reduced form, as illustrated in Table (ii). These include Peaks A and B, which correspond to α-SI (m/z 1357.58), and α-OI (m/z 1400.53), respectively, which are the focus of this study.

## Slide 3
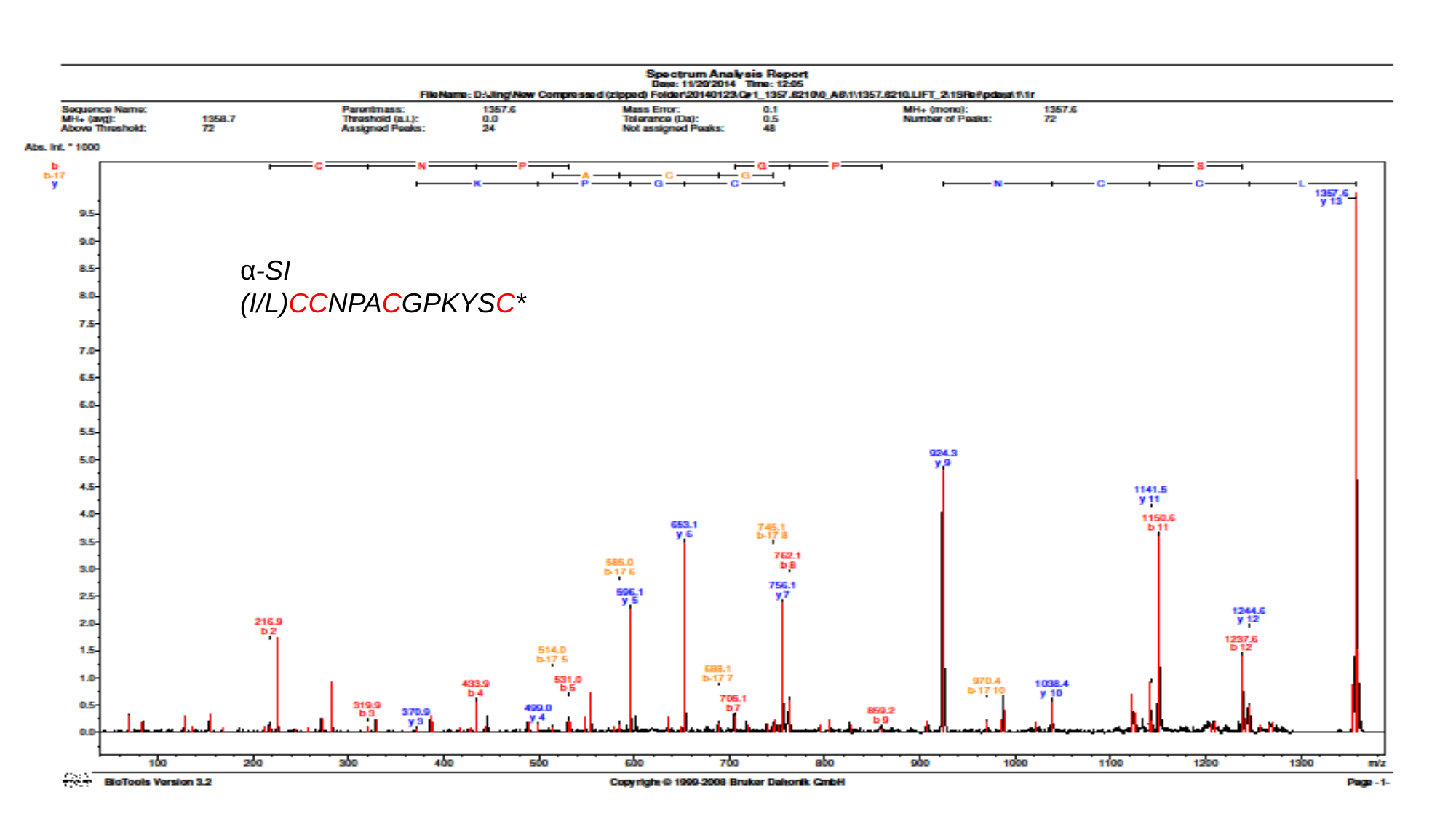

α-SI
(I/L)CCNPACGPKYSC*

## Slide 4
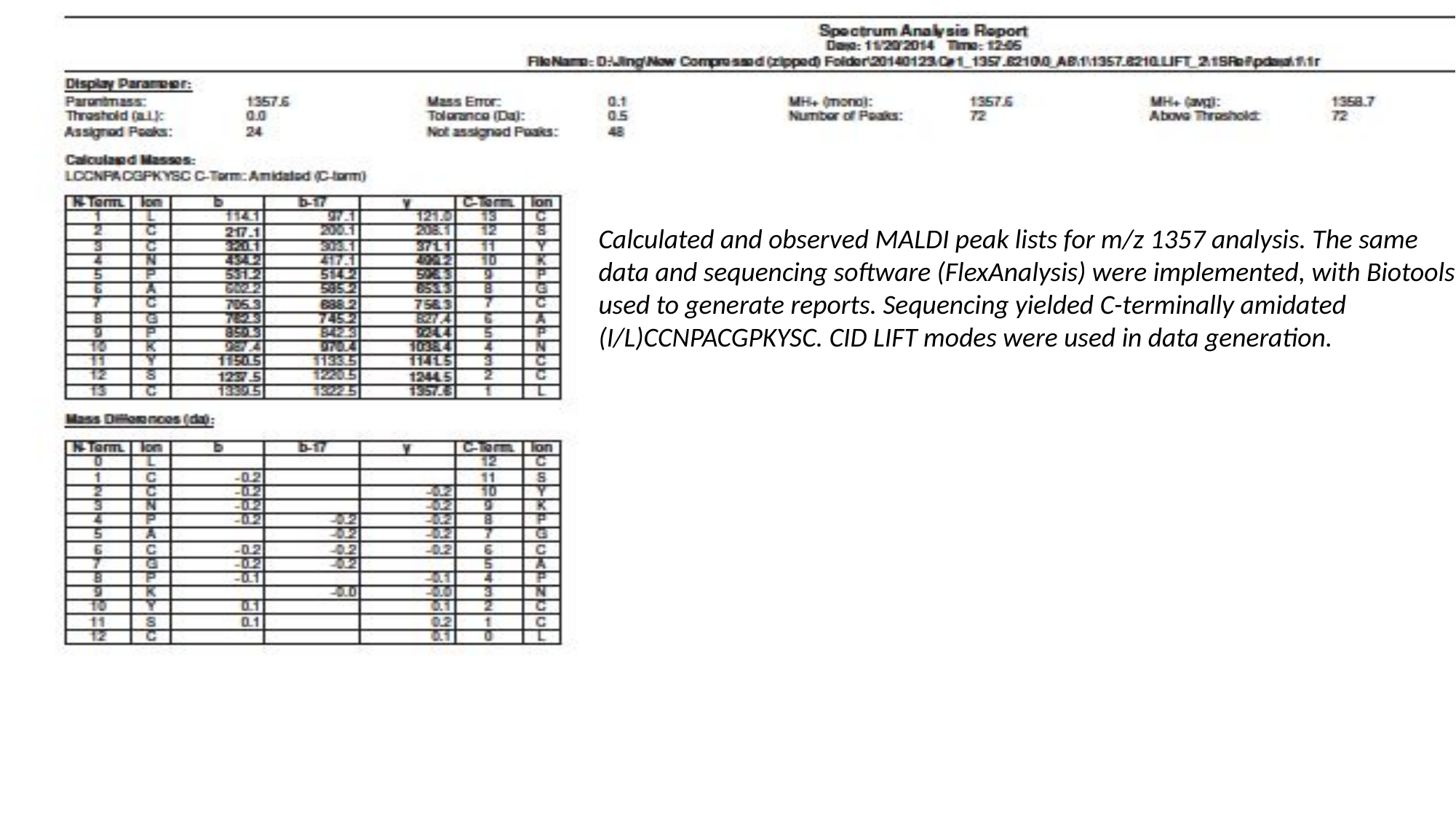

Calculated and observed MALDI peak lists for m/z 1357 analysis. The same data and sequencing software (FlexAnalysis) were implemented, with Biotools used to generate reports. Sequencing yielded C-terminally amidated (I/L)CCNPACGPKYSC. CID LIFT modes were used in data generation.

## Slide 5
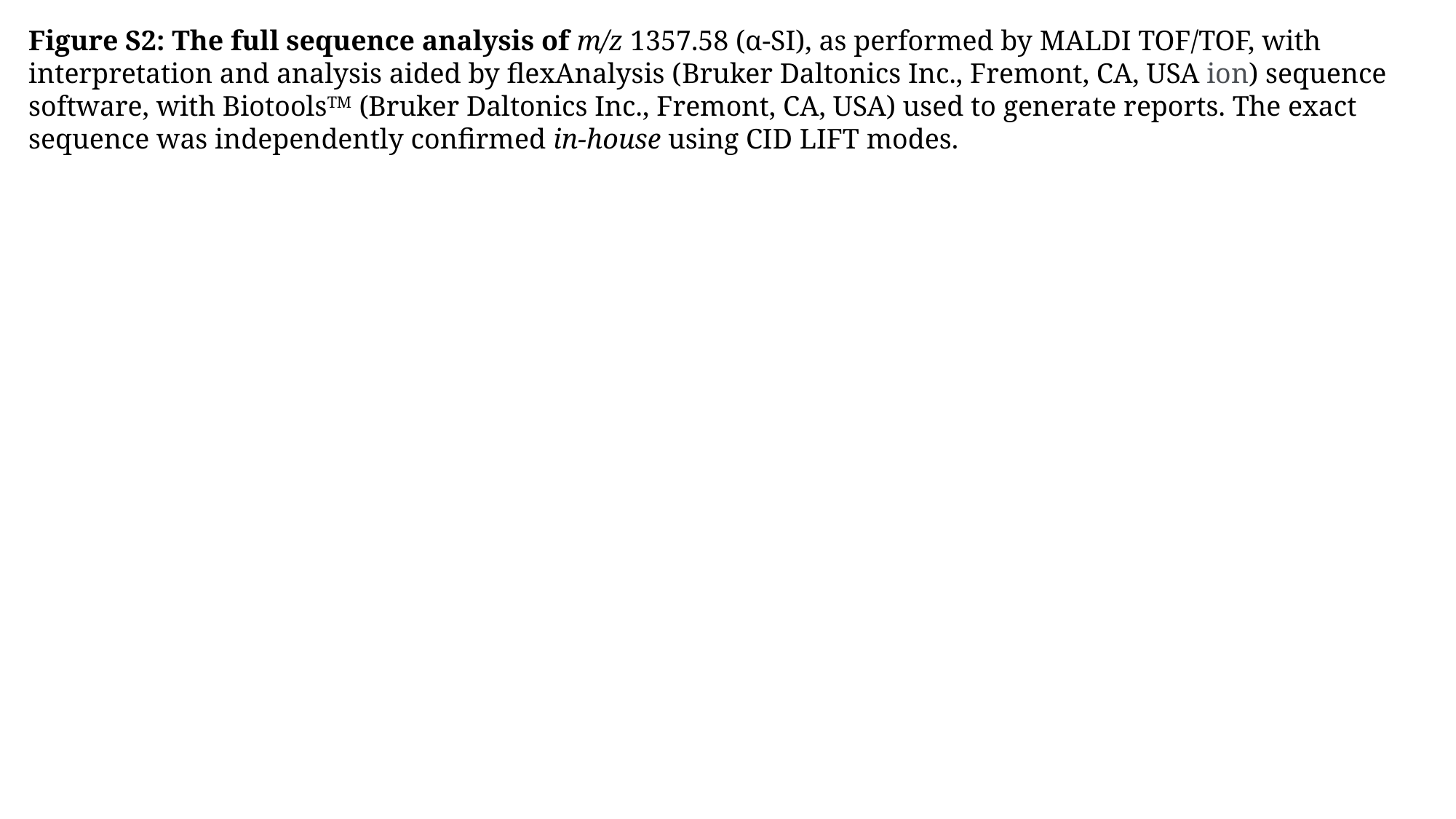

Figure S2: The full sequence analysis of m/z 1357.58 (α-SI), as performed by MALDI TOF/TOF, with interpretation and analysis aided by flexAnalysis (Bruker Daltonics Inc., Fremont, CA, USA ion) sequence software, with BiotoolsTM (Bruker Daltonics Inc., Fremont, CA, USA) used to generate reports. The exact sequence was independently confirmed in-house using CID LIFT modes.

## Slide 6
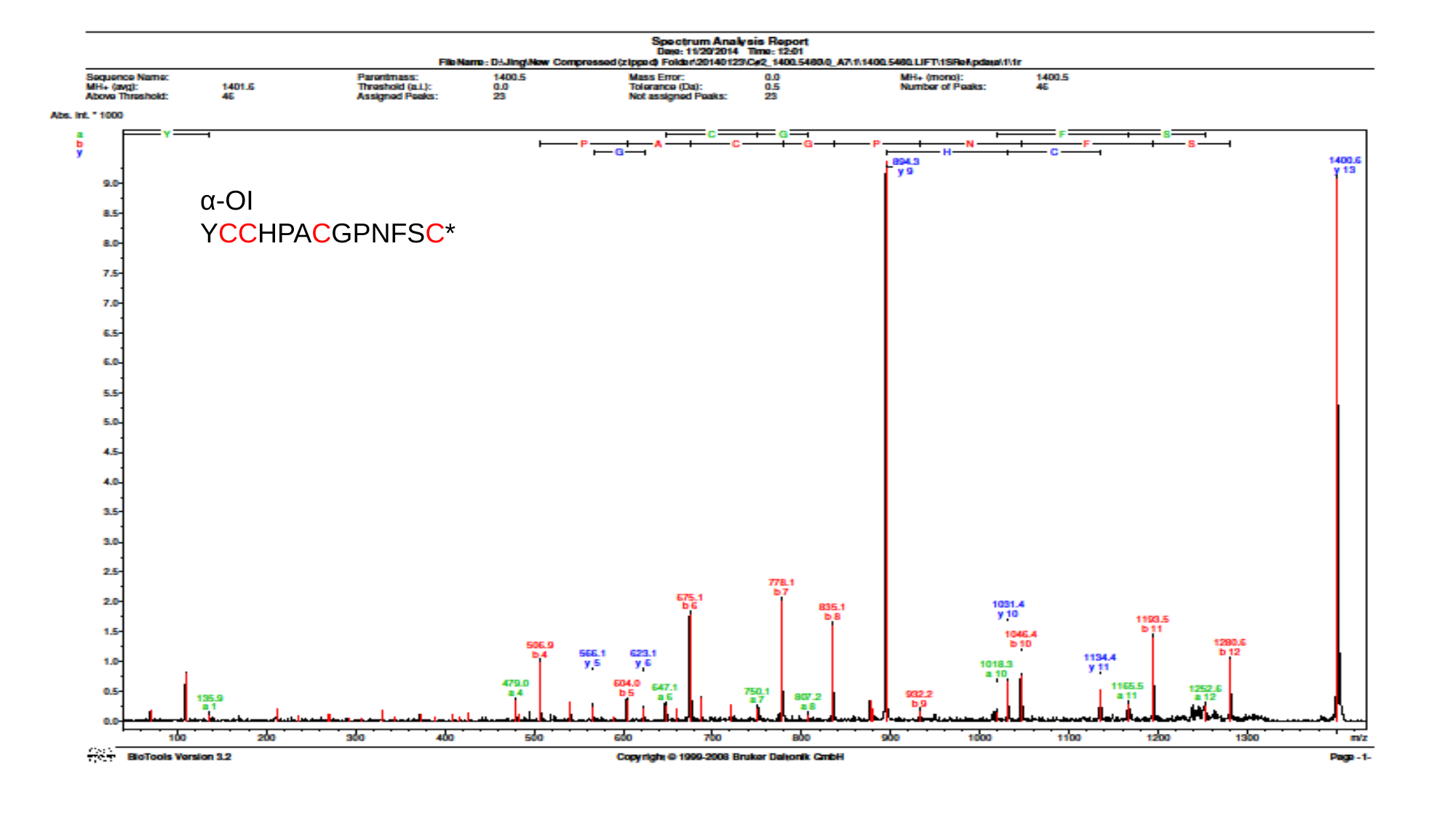

α-OI
YCCHPACGPNFSC*

## Slide 7
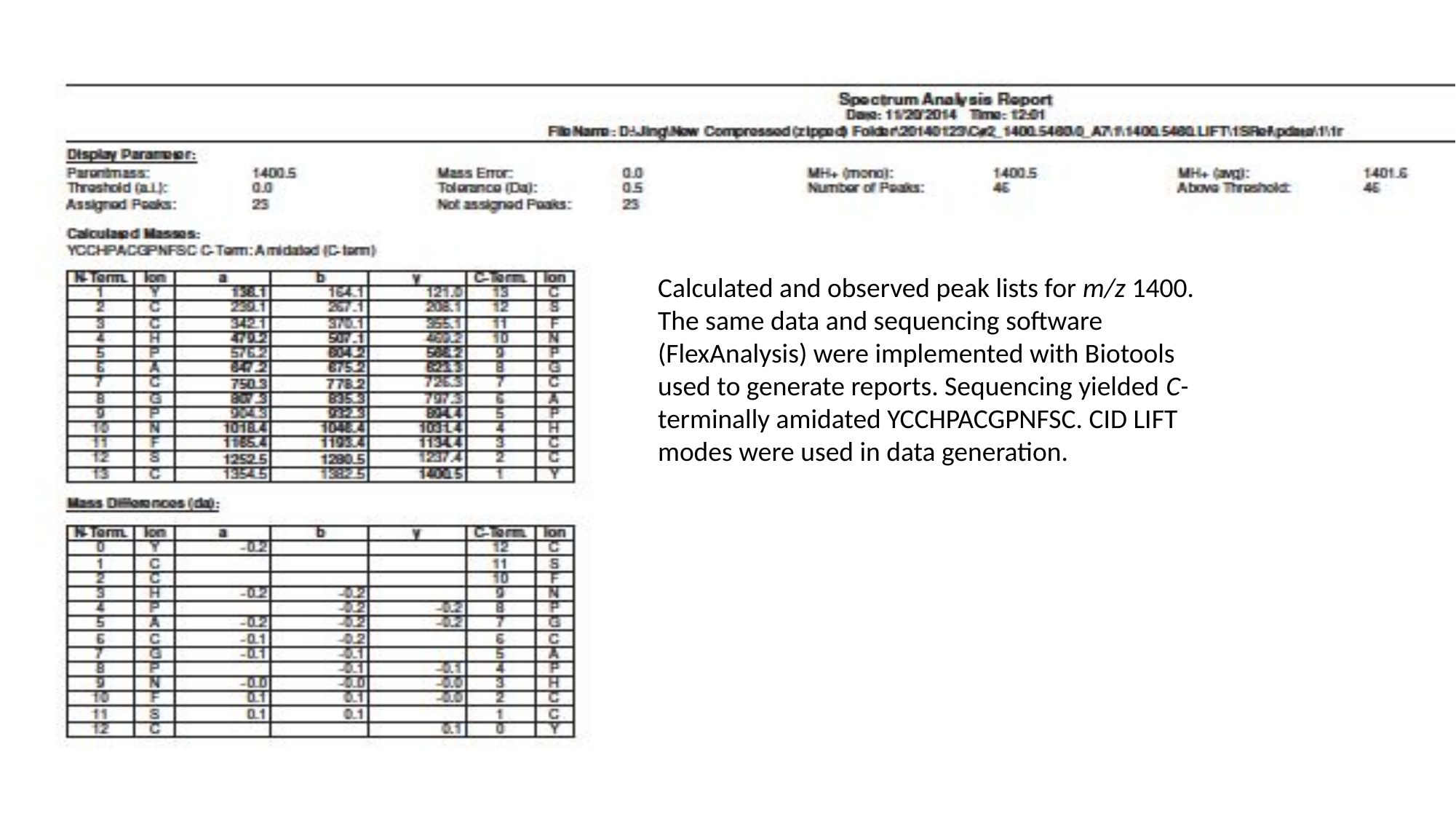

#
Calculated and observed peak lists for m/z 1400. The same data and sequencing software (FlexAnalysis) were implemented with Biotools used to generate reports. Sequencing yielded C-terminally amidated YCCHPACGPNFSC. CID LIFT modes were used in data generation.

## Slide 8
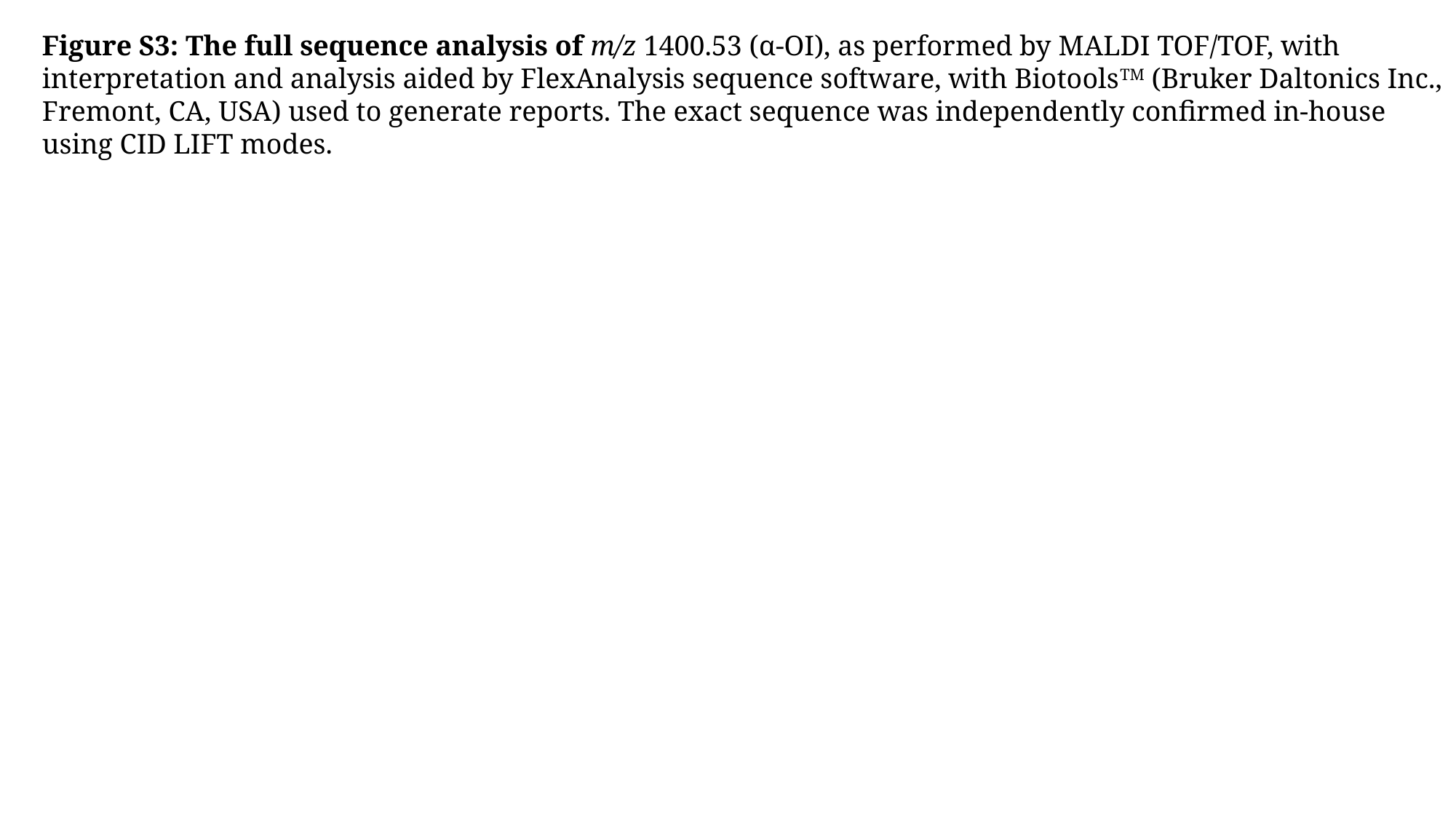

Figure S3: The full sequence analysis of m/z 1400.53 (α-OI), as performed by MALDI TOF/TOF, with interpretation and analysis aided by FlexAnalysis sequence software, with BiotoolsTM (Bruker Daltonics Inc., Fremont, CA, USA) used to generate reports. The exact sequence was independently confirmed in-house using CID LIFT modes.

## Slide 9
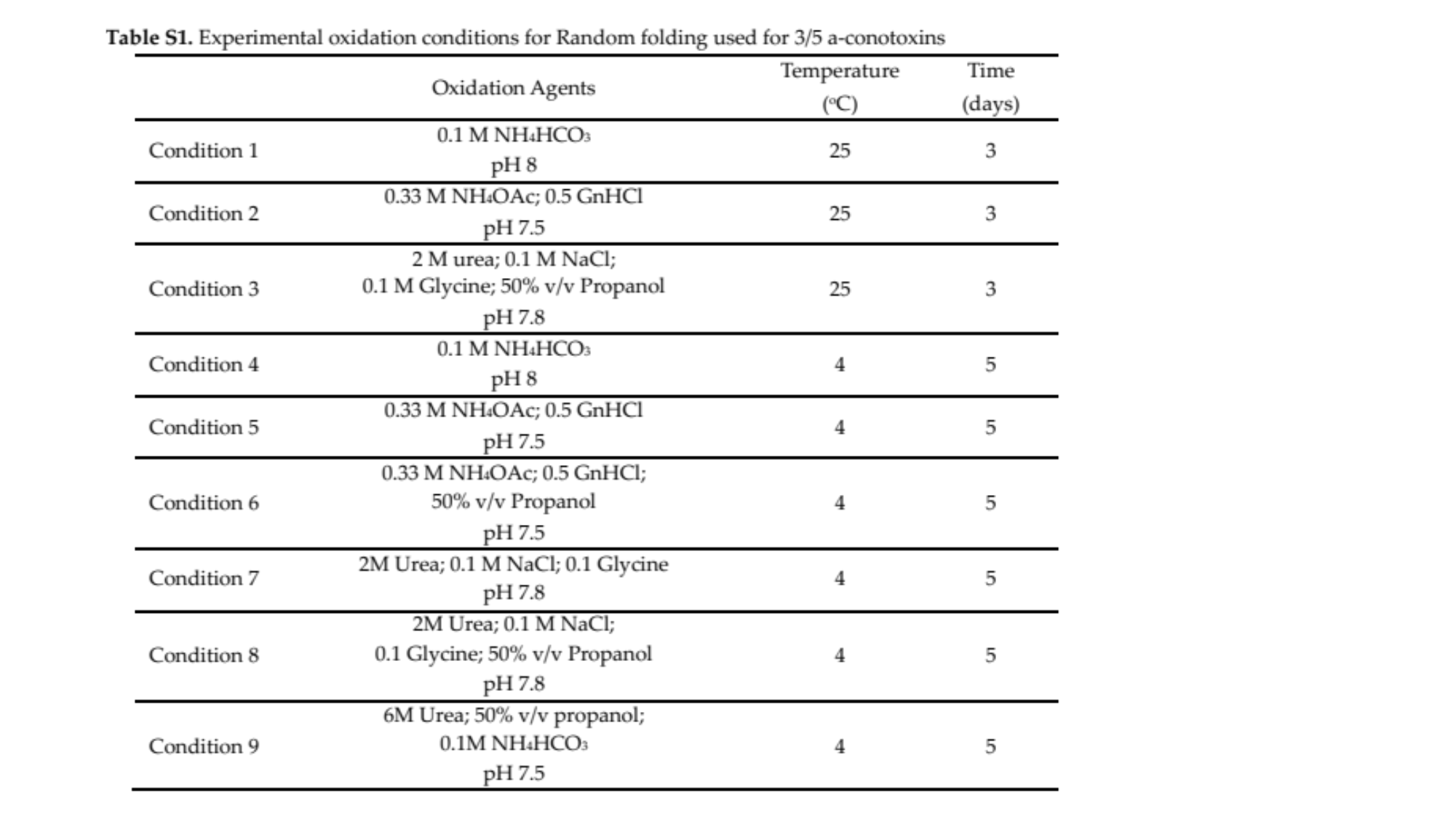

Supplement: Supplementary file 1 [file ijms-23-12096-s001.zip › ijms-1930868-supplementary.pptx]
